# Adipo-myokine Irisin, a Promising Antioxidant against Nicotine Induced Oxidative Stress in BALB/c mice

**DOI:** 10.12669/pjms.40.1.8140

**Published:** 2024

**Authors:** Madiha Imran, Sadia Ahsin, Gule-Naghma Saeed, Hira Ashraf

**Affiliations:** 1 Madiha Imran, MBBS, MPhil, ACMed, FCPS. Associate Professor of Physiology, Foundation University Islamabad, Islamabad, Pakistan; 2 Sadia Ahsin, MBBS, FCPS, MHPE. Professor and Head of Department of Physiology, Foundation University Islamabad, Islamabad, Pakistan; 3 Gule-Naghma Saeed, MBBS, MPhil, ACMed, FCPS. Professor of Physiology, Foundation University Islamabad, Islamabad, Pakistan; 4 Hira Ashraf, MBBS, MPhil, ACMed. Assistant Professor of Physiology, Foundation University Islamabad, Islamabad, Pakistan

**Keywords:** Irisin, Oxidative stress, Antioxidant enzymes, Lipid peroxidation, Nicotine

## Abstract

**Objectives:**

To determine the role of r-irisin in attenuating nicotine-induced oxidative stress by estimating serum oxidative stress markers and antioxidant enzymes in BALB/c mice.

**Method:**

This 18 month experimental study was carried out at Foundation University Islamabad and National Institute of Health starting in 2020. Thirty healthy BALB/c mice were divided into three groups. Group-I (control group) received normal saline 1ml/kg body weight intra-peritoneally daily for 28 days. Experimental group, Group-II received nicotine 2mg/kg body weight intra-peritoneally, for 28 days to induce oxidative stress. Experimental Group-III was given r-irisin 0.5 μg/g body weight/day via tail vein injection, for the last 14 days in addition to intraperitoneal nicotine for 28 days. On 29^th^ day, intra-cardiac blood samples were taken for estimation of serum antioxidant enzymes [Superoxide dismutase (SOD), Reduced Glutathione (GSH) and Catalases (CAT)], and Thiobarbituric Acid-Reactive Substances (TBARS) levels as lipid peroxidation marker using ELISA. SPSS version 24 was used for statistical analysis. Significant difference in parameters across groups was calculated using one way ANOVA. *P*-value of < 0.05 was considered significant.

**Results:**

Group-II showed statistically significant increase in serum lipid peroxidation marker (TBARS) levels (p*<0.001*) and reduction in serum anti-oxidative enzymes (SOD, CAT, GSH) levels (p*< 0.001*) as compared to Group-I. In Group-III, with co administration of r-irisin, significant improvement in antioxidant enzymes levels and reduction in TBARS levels was observed (p*< 0.001*) as compared to Group-II.

**Conclusion:**

Irisin ameliorates nicotine induced oxidative stress by improving serum anti-oxidant enzyme levels and reducing serum lipid peroxidation marker.

## INTRODUCTION

Oxidative stress, characterized by enhanced production of Reactive Oxygen Species (ROS) and decreased antioxidant defense mechanism, is the leading cause of disturbance in cellular homeostasis.^1^,^2^ Relative excess of free radicals has a number of detrimental effects on cellular and tissue functions and contributes in pathogenesis of various cardiovascular & metabolic diseases.^3^ Although these pathologies have multifactorial etiologies, oxidative stress plays a critical role in altering signaling pathways that are vital to maintain cellular homeostasis.^4^ ROS trigger cascade of pro-inflammatory mechanisms which lead to micro-vascular damage and subsequent organ dysfunction.

Under physiological conditions, an effective balance exists between pro-oxidant and antioxidant system. Several factors such as age, genetic makeup, diet, individual behavior and external stressors attribute to disruption of this balance.^5^,^6^ In healthy state, increase mitochondrial biogenesis and up-regulation of mitochondrial antioxidant system neutralize over production and cytotoxic effects of ROS. These efficient antioxidants enzymes reduce oxidative stress by scavenging ROS and restore homeostasis by minimizing inflammatory response, hence ameliorate oxidative stress induced tissue damage.^1^,^2^ Data from animal and human studies support the role of various supplements and an efficient antioxidant enzyme system in reverting detrimental effects of ROS on cellular and tissue functions. Reduction in activity of antioxidant enzymes such as SOD, catalase, and glutathione is positively associated with deleterious effects of reactive oxygen species in pathogenesis of a wide variety of disease states.^3^,^7^,^8^

Irisin, a peptide hormone, is secreted by skeletal muscle and adipose tissue in response to exercise due to proteolytic breakdown of fibronectin type III (FNDC5), a transmembrane protein.^9^ It has been postulated that irisin improves pro-oxidant-antioxidant balance by increasing mitochondrial enzymes expression.^6^ Recently, Irisin has gained great interest as potential target to combat diabetes, obesity and various metabolic disorders associated with these diseases. Literature has shown that irisin alleviates oxidative stress, induced due to diabetes mellitus, obesity and metabolic syndrome, by increasing antioxidant enzymes levels, and by increasing mitochondrial oxygen consumption and reducing intracellular ROS production.^10^-^12^

Irisin has been shown to exhibit protective effect against oxidative stress on cardiac myocytes and vascular tissue by improving mitochondrial functions in ischemia-reperfusion model.^6^ Irisin, therefore may serve as a link between exercise and its health benefits in various diseases. However, further studies are required for better understanding of anti-oxidative nature of irisin in mitigating adverse effects of nicotine induced oxidative stress.

Nicotine, a naturally-occurring alkaloid, found mainly in tobacco, is frequently absorbed from cigarette smoke.^13^ It is present in smokeless tobacco (vape and e-cigarette), smoking cessation medications and is an integral part of insecticides, Therefore, is a major source of accidental or intentional nicotine poisoning.^14^ Literature has revealed prolonged administration of nicotine at a steady nonlethal low dose enhances production of ROS (superoxide and other reactive oxygen species such as hydroxyl radical, hydrogen peroxide, and peroxy-nitrite) by disrupting mitochondrial respiratory chain. These ROS are capable of initiating and promoting oxidative damage by increasing lipid peroxidation and by decreasing or depleting mitochondrial antioxidant enzymes, such as catalases, glutathione and superoxide dismutase,^13^,^14^ therefore, the current study was designed to evaluate antioxidant effects of exogenous irisin in improving redox state by assessing antioxidant enzymes levels in nicotine induced oxidative stress in BALB/c mice. It is hypothesized that parenteral administration of irisin may protect against nicotine-induced oxidative stress, by increasing antioxidant enzyme levels in animals exposed to nicotine.

## METHODS

An experimental study was carried out from Jan 2020 to June 2021 at Department of Physiology, Foundation University Islamabad (FUI), Islamabad in collaboration with NIH (National Institute of Health).

### Ethical Approval:

Formal approval was obtained from Research Evaluation Unit (REU) (16^th^ May, 2018) of CPSP Islamabad and Ethical Review Committee (ERC) of FUI (26^th^ Oct, 2017 letter no; 217/FF/FUMC/ERC).

G* power method was used for calculating sample size. Thirty healthy, non-diabetic male BALB/c mice weighing 22-25 grams were obtained from NIH and were divided into three groups of ten each. The animals were housed in labelled standard cages in batches of five and were kept in properly ventilated and controlled temperature conditions. They were exposed to a 12-hours light/dark cycle and were given normal diet ad libitum. The diet was prepared according to the standards laid down by the Universities Federation for Animals Welfare and ARRIVE guidelines for animal research.^15^

Group I (control group) mice were given normal saline 1ml/kg body weight via intra-peritoneal injection daily for 28 days. Group II (nicotine group) mice were given nicotine (Alfa Aesar, Johnson Matthey Company, Great Britain) 2mg/kg body weight via intra-peritoneal injection daily for 28 days to induce oxidative stress.^13^,^14^ While group III (nicotine+r-irisin) mice, received r- irisin (0.5 μg/g bodyweight/day) for the last 14 days via tail vein injection obtained from Abbexa Ltd Cambridge, UK in addition to 2mg/kg body weight intraperitoneal nicotine for 28 day.^16^-^18^ On 29th day, mice were euthanized and terminal intra-cardiac blood sampling was done.

Blood samples were centrifuged at 4000 rpm for 20 minutes for the separation of serum. Supernatant was pipetted out and was used for analysis of lipid peroxidation marker: Thiobarbituric Acid-Reactive Substances TBARS and serum antioxidant enzyme levels [Superoxide dismutase (SOD), Reduced Glutathione (GSH) and catalases (CAT)] by using double antibody sandwich ELISA (Enzyme-Linked Immunosorbent Assay). Mouse ELISA kits for SOD, CAT, GSH and TBARS (Catalogue No: 20684, 20583, 20576 and 21479 respectively) were obtained from Glory Science Co., Ltd (Taiwan). The absorbance at 450 nm was read within 15 minutes of adding the stop solution. Standard calibration curves were plotted on graph paper with absorbance (OD) on y axis and standard concentrations of each antioxidant enzymes and lipid peroxidation marker on x axis.

SPSS version 24 was used for statistical analysis. Values were expressed as means ± SD. One way ANOVA was used to calculate significant difference in parameters across groups followed by Tukey’s HSD (Honestly Significant Difference) post hoc test for multiple comparisons among the groups. A *p*-value of < 0.05 was considered statistically significant.

## RESULTS

**Fig.1:**
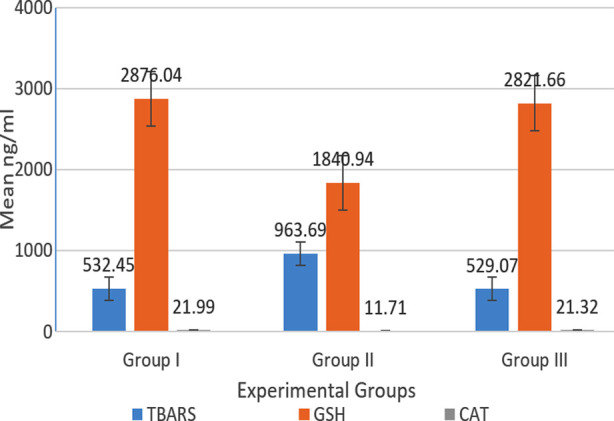
Comparison of mean values of Serum TBARS, glutathione reductase (GSH) and catalase (CAT) among the three groups. Group I (control), Group II (nicotine), Group III (nicotine+r-irisin).

Effect of nicotine & r-irisin administration on antioxidant enzymes and lipid peroxidation markers is shown in Table-I & II. The results are expressed as means ± SD, (Fig.1). Oxidative stress was evident with significant increase in lipid peroxidation marker (TBARS) levels in Group-II (Nicotine group), as compared to Group-I (control group) *(p<0.001)*. Statistically significant decrease in mean serum antioxidant enzymes levels (SOD, CAT and GSH) was seen in Group II, as compared to the Group-I *(p<0.001)*.

**Table-I:** Comparison of serum superoxide dismutase (SOD), catalase (CAT) glutathione reductase (GSH), and TBARS levels among the three study groups.

Groups	Variables			

	Serum SOD pg/ml	Serum CAT ng/ml	Serum GSH ng/ml	Serum TBARS ng/ml
Group-I (n=10)	5866.48± 60.42	21.99± 0.42	2876.04± 86.52	532.45 ±12.38
Group-II (n=10)	4967.57± 19.23	11.71± 0.27	1840.94± 24.79	963.69± 40.25
Group-III (n=10)	5793.47±101.41	21.32± 0.58	2821.66± 50.72	529.07± 18.96
p-value	<0.001*	<0.001*	<0.001*	<0.001*

The results are means SD.

* p ≤ 0*.*05 was considered significant. The group means were compared using ANOVA. Group-I (control), Group-II (nicotine), Group-III (nicotine+r-irisin).

**Table-II:** Pairwise comparison of serum superoxide dismutase (SOD), catalase (CAT) glutathione reductase (GSH) and TBARS levels among the three study groups.

Pairwise Comparison of Groups	Variables			

	Serum SOD pg/ml	Serum CAT ng/ml	Serum GSH ng/ml	Serum TBARS ng/ml
Group I vs Group II	<0.001*	<0.001*	<0.001*	<0.001*
Group I vs Group III	0.09	0.07	0.12	0.95
Group II vs Group III	<0.001*	<0.001*	<0.001*	<0.001*

* p ≤ 0*.*05 was considered significant. Pairwise comparison between the groups was done using post hoc Tukey’s HSD test. Group-I (control), Group-II (nicotine), Group-III (nicotine+r-irisin).

In Group III (nicotine+ r-irisin group) in comparison to Group II, significant improvement in mean serum anti-oxidant enzymes levels (SOD, CAT and GSH) *(p<0.001)* was seen along with statistically significant reduction in oxidative stress marker levels (TBARS) with administration of r-irisin *(p<0.001)*.

Post hoc analysis showed statistically significant difference between Group-II and III however, no significant difference was seen between Group-I and Group-III in oxidative stress markers and antioxidant enzymes levels.

## DISCUSSION

Regular and moderate physical exercise promotes health. Irisin, also known as “exercise hormone” released during moderate aerobic endurance activity,^19^ has shown to exhibit many beneficial effects in treatment of cardiovascular diseases, diabetes, hyperlipidemia and smoking.^17^,^19^ Results of current study showed administration of r-irisin attenuated nicotine-induced oxidative stress by up-regulating serum antioxidant enzymes levels, thus establishing the protective role of irisin (Table-I).

Outcomes of our research have been confirmed by Wang et al, and Bi et al, who demonstrated protective role of irisin in alleviating oxidative stress using ischemia/reperfusion (I/R) injury model. Both studies proposed parenteral administration of irisin enhanced mitochondrial biogenesis, with subsequent reduction in oxidative stress markers (MDA) and increase in antioxidant enzyme levels (SOD and GSH-Px).^20^,^21^ Likewise, Ren et al., established antioxidant nature of irisin in intestinal tissues by observing significant reduction in intestinal Malondialdehyde (MDA) levels and improvement in SOD and GSH levels with parenteral Irisin administration.^22^ Similarly, et al., highlighted the potential antioxidant function of irisin in protecting the heart from ischemia and reperfusion injury. Administration of irisin led to improved mitochondrial function, increased expression of SOD and a decrease in the size of myocardial infarctions.^23^ These findings were in line with current study where significant decrease in oxidative stress markers and improvement in antioxidant enzymes level (SOD, CAT and GSH) was observed, thereby reinforcing protective nature of irisin in oxidative stress.

Clinical studies have shown association between oxidative stress and nicotine intake. Nicotine, a major constituent of tobacco products and insecticides, is known to impair redox status due to accumulation of lipid peroxidation products and reduction in activity of the major antioxidant enzymes in plasma of smokers.^13^ In current study, nicotine administration in experimental groups, increased production of ROS which resulted in oxidative stress as evident by increased lipid peroxidation and decreased activity of SOD, glutathione reductases and catalases highlighting the potential toxic effects of nicotine. Studies have shown use of Rosella supplementation and fenofibrate in improving the redox state in nicotine induced oxidative stress, however, role of Irisin in nicotine induced oxidative stress is not much explored.^13^,^14^

Sajid F et al., evaluated the effect of smokeless dipping tobacco (Naswar) on antioxidant enzymes glutathione per oxidase (GPx), SOD and lipid profile. Use of naswar showed injurious effects on health, manifesting as disruptions in lipid profiles and notable reductions in levels of crucial antioxidant enzymes.^24^ Studies have shown wide spread detrimental effects of nicotine on other tissues as well. Jabeen A et al., and Kiyani HP et al., studied nicotine-induced oxidative stress in renal and hepatic tissues which was mitigated by ghrelin, a naturally occurring peptide hormone. Nicotine toxicity was evident by raised hepatic and renal oxidative stress markers (MDA) and decreased antioxidant enzymes (such as SOD, CAT, and GSH), as also observed in current study.^25^,^26^

Literature has shown irisin administration improved oxidative status in various metabolic disorders.^27^ Zhu et al, and Schaalan et al, in two separate studies established anti-oxidative, anti-inflammatory, and anti-obesity properties of endogenous irisin in their studies. They studied role of exercise induced irisin release in attenuating diabetes induced oxidative stress on vascular tissue and in obesity. Their conclusions highlighted that exercise training increased endogenous Irisin production which played a substantial role in lowering body weight, improving dyslipidemia, reducing lipid peroxidation markers, and enhancing total antioxidant capacity. They further suggested that exercise resulted in increase in irisin levels by increasing fibronectin type III domain-containing protein 5(FNDC5) gene expression thereby relieving oxidative stress.^18^,^28^ Effect of r- irisin on tissue oxidative stress and aortic tissue endothelial dysfunction was evaluated by Sarwar M et al., and they established that exogenous irisin ameliorates nicotine-induced oxidative stress and vascular dysfunction by improving antioxidant enzymes’ levels with corresponding decrease in membrane lipid peroxidation and severity of aortic tissue damage.^29^

In current study, exogenous irisin administration was preferred over naturally released exercise induced irisin to mitigate nicotine induced oxidative stress. Research in the literature has demonstrated that irisin is naturally released from muscles in response to physical exercise. However, the exact duration and intensity of exercise that would produce enough irisin to mitigate oxidative stress has not been documented. The current study with calculated standardized dose of irisin and nicotine gives baseline for future projects to establish minimum duration and intensity of exercise which would be required to produce desired antioxidant effect of irisin. Exercise-induced irisin release could present a promising therapeutic approach for preventing vascular injuries caused by smoking and harmful effects of nicotine from other sources like vape and e-cigarette. Further research in this direction could provide valuable insights into the potential benefits of irisin as a preventive measure.

### Limitation

Due to limited time and resources, mitochondrial signaling pathways involved in nicotine induced oxidative stress could not be assessed.

## CONCLUSION

Based on findings of this study it can be concluded that irisin treatment improves nicotine-induced oxidative stress evident by serum oxidative stress markers, and antioxidant enzyme levels assays. This inference can be effectively utilized to counter the oxidative stress induced by direct or indirect intoxication with nicotine via active or passive smoking and/or use of insecticides.

### Recommendation

Further studies are required to understand molecular and genetic control of irisin regulation and its potential therapeutic role.

### Author’s Contributions:

**MI:** Main author, conception, sampling, writing and also responsible for the integrity and accuracy of the manuscript.

**SA:** Supervised research, conception, review.

**GNS:** Co supervision, data analysis, review.

**HA:** Data analysis, proof reading, assistance in sampling.
